# Key performance indicators and benchmarks in MCI prehospital response using technological tools: a qualitative study assessing the perception of practitioners and tool developers

**DOI:** 10.1007/s00068-024-02627-3

**Published:** 2024-08-22

**Authors:** Hamdi Lamine, Nikolaos Markou-Pappas, Luca Ragazzoni, Marta Caviglia

**Affiliations:** 1 CRIMEDIM – Center for Research and Training in Disaster Medicine, Humanitarian Aid and GlobalHealth, Università del Piemonte Orientale IT, Vercelli, Italy; 2 Department for Sustainable Development and Ecological Transition, Università del Piemonte OrientaleIT, Vercelli, Italy; 3 Department of Translational Medicine, Università del Piemonte Orientale IT, Vercelli, Italy; 4 Department of Community Health Nursing, College of Nursing, University of Hail, Hail, Saudi Arabia

**Keywords:** Mass Casualty Incident, Prehospital care, Performance evaluation, Key performance indicators, Technology, Qualitative

## Abstract

**Purpose:**

The aim of this study is to investigate the opinions and perspectives of The Novel Integrated Toolkit for Enhanced Prehospital Life Support and Triage in Challenging and Large Emergencies (NIGHTINGALE) end-users and tool developers regarding Key Performance Indicators (KPIs) and benchmarks that assess the prehospital response to Mass Casualty Incidents (MCIs) enhanced by the NIT-MR.

**Methods:**

A qualitative study employing focus group discussions was conducted to collect opinions and perspectives of end-users and tool developers regarding KPIs and benchmarks in MCI response using the NIT-MR. The criteria considered for the selection and distribution of participants within the groups was the nature of their involvement within the NIGHTINGALE project and their familiarity with the tools to be discussed.

**Results:**

Thirty-one participants from different countries were included. Four themes emerged during data analysis which are: definition/explanation is the personal understanding of participants of the term KPI, process of KPI development and relationship with User Requirements is the decision process for assigning KPIs to user requirements, benchmarking is the mental process of associating a benchmark to a KPI or for developing a benchmark, and technical/medical gap is the gap of understanding between each sides’ fields.

**Conclusion:**

This study emphasized the need for a structured approach to using KPIs and bridging the gap between technological and medical worlds, taking the NIGHTINGALE project, funded by the European Union, which aims to develop a technological toolkit for first responders in mass casualty incidents as an example. These insights are crucial for enhancing disaster response.

**Supplementary Information:**

The online version contains supplementary material available at 10.1007/s00068-024-02627-3.

## Background

In recent years, there has been a growing interest in researching and developing technological tools to aid first responders (FRs) in sudden onset disasters and mass casualty incidents (MCIs). In alignment with this trend, the European Union (EU) has funded the ‘Novel Integrated Toolkit for Enhanced Prehospital Life Support and Triage in Challenging and Large Emergencies’ (NIGHTINGALE) project. This initiative seeks to bolster the preparedness and efficiency of first responders (FRs) by fostering collaboration among various organizations and providing technological solutions capable of supporting triage, prehospital life support, damage control interventions, and prehospital processes during MCIs [1, 2]. The NIGHTINGALE project brings together tool developers and end-users, including medical and non-medical practitioners, scientific medical societies, and academic research centers [3]. The goal of NIGHTINGALE is to create a ‘Novel Integrated Toolkit for emergency Medical Response’ (NIT-MR). This comprehensive toolkit encompasses interconnected wearable technologies, sensors, mobile applications, unmanned aerial vehicles, and coordination systems that facilitate real-time multi-agency crisis management operations, along with artificial intelligence prediction tools [1]. The development of NIT-MR was guided by a comprehensive set of end-user requirements (UReqs) categorized according to the MoSCoW prioritization model [4], and identified through an iterative process to ensure alignment with the end-users’ needs and broader project objectives.

As the next step, the NIGHTINGALE project plans to test the NIT-MR solution through various trials and assess its impact on FRs’ performance using a series of key performance indicators (KPIs) [1, 5].

KPIs are quantifiable metrics employed for evaluating performance, monitoring progress and pinpointing areas that may need improvement. Additionally, they facilitate the measurement on how well UReqs are fulfilled, establishing a feedback loop for informed decision-making regarding product enhancement aimed at better alignment with needs. To provide an example, if a UReqs stresses the importance of rapid response times in the context of a new technology introduced in the MCI response, a relevant KPI could be “average response time” to evaluate the fulfillment of this requirement and the impact of the technology in the FRs’ performance. While various frameworks exist for developing KPIs in healthcare systems and healthcare technologies [6–9], the unique context of prehospital response to MCIs presents distinct challenges due to the unpredictable nature of such situations, the dissonance between contingency plans and reality, and frequent communication failures [10]. Furthermore, the lack of standardization in the structure and function of prehospital emergency medical services (EMS) worldwide leads to variations in protocols, procedures, and communication practices, hindering the ability to evaluate and compare the effectiveness of different response strategies across regions and jurisdictions [11, 12]. Given that the future of responding to sudden onset disasters and MCIs increasingly rely on technological solutions, quantitatively assessing the performance of these tools is of paramount importance. Investigating the current gaps and barriers in identifying specific quantitative KPIs provides an opportunity to suggest potential solutions. Therefore, this study aims to explore the opinions and perspectives of NIGHTINGALE end-users and tool developers regarding KPIs and benchmarks for assessing the prehospital response to MCIs enhanced by NIT-MR.

## Methods

A qualitative study employing focus group discussions (FGDs) was conducted to collect opinions and perspectives of end-users and tool developers regarding KPIs and benchmarks in MCI response using the NIT-MR. This design was chosen because of the exploratory nature of the study and because it can properly document the experiences of the participants and generate discussion points [13, 14]. Methods have been reported in accordance with the Consolidated Criteria for Reporting Qualitative Research (COrEQ) [15]. The criteria considered for the selection and distribution of participants within the groups was the nature of their involvement within the NIGHTINGALE project (tool developer and end-users) and their familiarity with the MCI prehospital process to which each specific tool of the NIT-MR would be applicable (eg. Prehospital triage, tactical coordination, performance of life saving interventions, …).

### Data collection

A FGD guide including aims, probing questions, and tasks for the moderators was elaborated, based on the overall objective of extracting a series of quantitative KPIs and related benchmarks from UReqs previously elaborated for each of the NIT-MR tool. Three FGDs were conducted simultaneously. Each group was nearly equally divided between end-users and tool developers and was led by one of the end-users. Each group had 4 (± 1) tools to discuss (Table 1). A presentation of the goal of the FGDs was made prior to starting the discussions, including definitions for KPIs and benchmarks. Additionally, a short document summarizing the main functionalities of the tools was distributed. FGDs were conducted on the 25th of October 2022, and each lasted approximately 2 h (± 15 min). After receiving written consent through Google Forms from the respondents, an audio recording was made for each FGD, and manual notes were taken by the moderators.

**Table 1:** Category of Novel Integrated Toolkit for emergency Medical Response (NIT-MR) tools assigned to the three groups

	Group A	Group B	Group C
Category of NIT-MR tools	Triage and vital signs devices	Interoperability and data fusion	UAV
	Multi agency collaboration systems	Command, control, and coordination management system	Damage control and AI-based diagnosis and prognosis

### Data analysis and reporting

The discussions of the 3 groups were recorded using smartphones, the recordings were transcribed manually (NMP), the transcripts were screened, cleaned, and read multiple times for accuracy (HL). Two researchers (HL, NMP) prepared a list of potential codes, using inductive reasoning, after reading all the transcripts and extracting the most important topics addressed by the participants. The two lists were then compared, and a unified codebook was devised to analyse the transcripts. The unified codebook was used to deductively code each of the transcripts, extracting significant quotes related to the generated codes. To ensure the consistency and reliability of the coding, both researchers were present. A time of approximately four hours was allocated for the analysis of each transcript. The analysis was conducted over the course of one week and was done using Atlas.ti software.

### Ethical considerations

The study was conducted according to the principles enunciated in the Declaration of Helsinki. All participants were required to provide a written informed consent, using google forms, prior to data collection. Sufficient details about the study aims and processes were provided before the start of the focus groups discussions. The collected data were anonymized, and access to the data was restricted to the research team (HL, NMP, LR, MC).

## Results

Thirty-one participants from different countries (EU countries and outside EU) were included in this study. Each group was composed of 10 (± 2) participants (Table 2).

**Table 2:** Demographics of the participants to the FGDs

Participants	N (%)
Gender	
Female	6 (19)
Male	25 (81)
Occupation	
Tool developer	12 (39)
Medical Practitioner	11 (35)
Non-medical practitioner	3 (10)
Academic position (researcher/professor)	5 (16)
Total	31

A total of 4 themes emerged during data analysis. An overview of the themes identified and their explanation, and number of quotes is presented in Table 3.

**Table 3:** Themes identified during the focus group discussion

Codes	Explanation	Number of quotes identified
Definition/explanation	Definition that participants gave to the KPIs/understanding of the participants of the term KPIs	30
Process of KPI development and relationship with UReqs	Process of KPI development and the understanding of the decision process for assigning KPIs to user requirements	145
Benchmarking	Mental process of associating a benchmark to a KPI/developing a benchmark	74
Technical/medical gap	A gap of understanding between each sides’ fields	17

### Definition/explanation

Prior to commencing the discussion, a shared definition of KPIs has been introduced to all participants. This definition characterizes KPIs as ‘*metrics used by some organisations to track the success and guide their progress towards specific strategic objectives’* [16]. Despite the given definition, participants suggested that within this context of using new technologies in MCI management, a KPI is ‘*a measurable element that can reflect how MCI management is going to be ameliorated by the specific tool*’, thus focusing specifically on performance enhancement by the tool. As an example, instead of recommending KPIs focused on quantifying a specific process (such as using a time-based indicator), they suggested KPIs aimed at assessing the degree of improvement achieved in that particular process. Furthermore, as participants aimed to grasps KPIs more comprehensively, they categorized them into three primary groups (operational, absolute, and quality indicators).

### Process of KPI development and relationship with UReqs

The instruction that participants had to follow was to start from UReqs of the NIGHTINGALE tools and assign KPIs to each UReq. An example was given to them stating that from a UReq “Prompt dispatch of ambulances at the MCI scene” a KPI could be “Average response time of ambulances to an MCI scene”. Most of the participants agreed with this process that the extraction of KPIs starts from the UReqs, ‘*the general idea is to start reading the user requirements and from that user requirements come up with a series of KPIs*’, additionally, according to them, each UReqs can have only one KPI associated to it. Since UReqs have already been prioritized using MoSCoW system, participants associated the level of specificity of the KPI to the priority assigned to the requirement, ‘*further we go from the must, the more specific our KPIs have to be*’. However, some participants advocated shifting the focus towards the tools and their functionalities rather than the performance of FRs. Yet, this approach introduced complexities and limited the identification of these KPIs, since it focused on the tools themselves rather than the process they supported. Indeed, while UReqs served as the initial step for most in formulating specific KPIs (as instructed), some other participants proposed starting from the use cases of the tools, the evaluation activity (type of exercise), or the literature to initiate discussion and create the KPI. To help in identifying the proper indicator, participants chose a set of criteria that, according to them, ensure the quality of the KPI. Those criteria were simplicity, specificity, and flexibility (ability to be contextualized). While all participants acknowledged KPI development as a collaborative process, they emphasized its dependence on end-users more than on tool developers. Of note, many participants manifested difficulties of associating KPIs with certain tools such as AI based tools. A mapping of the KPIs extracted is provided in the supplementary material.

### Benchmarking

In a similar fashion to what occurred with KPIs, a definition of benchmarking was conveyed to the participants. This definition explained that benchmarking encompasses the establishment of a reference point for evaluating the performance of individual responders or the entire system. According to the participants, a benchmark needs to be context dependent and goal oriented, and must be categorized in one of three types: qualitative, quantitative, or technical (associated with tool performance). Benchmarks were identified through either agreed-upon suggestions or examples from previous experiences. Even though, both these worlds seek to measure and evaluate the effectiveness of the same processes or activities, they do so, using vastly different tools, methods, and criteria. While the use of data and performance metrics is increasingly becoming a part of medical practice, the gap between the two fields is still significant. Even though, the tools and their functionalities limited the identification of benchmarks (much like they did with KPIs), participants suggested that benchmarking should commence by a) gaining a deeper understanding of how the KPI can be most effectively measured, b) using real-life examples, or c) referring to a common point of reference from the literature.

### Technical/medical gap

During the FGDs, participants with diverse backgrounds were brought together, mainly comprising of medical practitioners and technical developers (Table 2). Even though this diversity was considered as an asset to the discussion, a gap in understanding each other’s respective backgrounds was observed. To bridge this gap, many attempts were made from both sides by giving simple examples understandable to someone from outside one’s field. One common thing in this interaction between medical and technical fields, is that, given the particularity of the NIGHTINGALE project, it was unclear to the participants whether the starting point of the KPIs development process should be medical or technical.

## Discussion

KPIs are commonly used to evaluate the efficiency of FRs’ performance in prehospital response [17]. This evaluation helps identify areas for improvement, guide decision-making, and monitor preparedness during training [18–20]. The NIGHTINGALE project, aiming to enhance prehospital life support and triage, faces challenges in standardizing the evaluation of training sessions and real-world MCI and disaster response. This study, which focused on exploring the opinions and perspectives of NIGHTINGALE end-users and tool developers regarding KPIs and benchmarks for assessing the prehospital response to MCIs enhanced by NIT-MR, uncovers some of the difficulties in achieving this standardization.

The lack in use of consistent terminology across the various phases of MCIs and disasters is an issue which persists [17, 18, 21–23]. The diverse group of experts involved in the NIGHTINGALE project demonstrated a difference in understanding of both the term and the usage of KPIs, highlighting the challenge of grasping the definition, role, utility, and use of performance indicators [18]. During the process of defining a KPI within the project’s framework, participants expressed their strong desire to leverage KPIs to quantify the improvements brought about by technology implemented in the context of prehospital MCI response. This sentiment reflects the widespread expectation among end-users that any technological tool introduced in the healthcare sector, particularly in critical situations like crises and emergencies, should deliver added value and measurable enhancements in performance. Essentially, there’s a common assumption that technology should inherently lead to improvements, especially in quantitative terms [24]. However, existing studies have highlighted scenarios where the opposite outcome may be observed [25]. In the context of technology-enhanced prehospital response to MCIs, the process of KPI identification presents a challenge. Several papers in the literature have highlighted the persistent difficulty of introducing multiple frameworks to standardize the identification of KPIs. The starting point for KPI identification is often an assessment of the key processes included in the prehospital response and consequently built and tailored upon [23, 26]. As a result, the KPIs identified and used to evaluate the performance of a specific process are deemed to have both their prospects and limitations dependent on the process they are designed to evaluate. This underscores the need for a more unified understanding of what KPIs are and what they are meant to achieve.

Indeed, while some participants were more concerned with objectively evaluating the FRs’ tasks, a subset of participants showed greatest interest in validating the tools to be used through KPIs.

This was likely due to the complexity and multidisciplinary nature of the project in question, which further highlights the need for a more unified understanding of what KPIs are and what they are meant to achieve.

Benchmarking, a critical aspect of KPI identification, is the process of setting a value against which individual responders or the overall system’s performance can be evaluated [17]. This process was found to be highly dependent on the country, system, context, and situation in question. This means that a benchmark for the same indicator may not be applicable in another country or in another context, due to different physical geography, resources, legal and regulatory frameworks, which emphasizes the importance of considering the specific circumstances within which KPIs are being developed and used [22].

The gap between the technological and medical worlds poses a significant challenge. The technological and medical worlds are two distinct realms with different approaches to problem-solving and decision-making, often having very different perspectives and priorities when it comes to KPI identification and evaluation [24, 27–29]. Even though both these worlds seek to measure and evaluate the effectiveness of the same processes or activities, they do so by using vastly different tools, methods, and criteria. While the use of data and performance metrics is increasingly becoming a part of medical practice, the gap between the two fields is still significant and may. This gap may be even more widened by the complexity of medical data and the challenges of integrating this data into technological systems, as well as the need for medical professionals to exercise judgment and make subjective decisions based on individual patient needs [24, 27–29].

The challenges identified in the NIGHTINGALE project align with previous research that has highlighted the perceptual gaps between different stakeholders in the healthcare and technology domains. For example, a study by Ndabu et al. observed perceptual gaps between clinicians and technologists on health information technology-related errors in hospitals. This study underscores the importance of addressing the differences in understanding and perception between different stakeholders to ensure the effective use of technology and performance indicators in healthcare settings [24].

## Recommendations

This study has provided important insights into the challenges of identifying KPIs and benchmarks that assess the prehospital response to MCIs supported by the NIT-MR. The participants in the FGDs demonstrated how urgent it is to develop criteria for assessing technology-enhanced disaster response efforts. Following are some recommendations drawn from these conclusions and discussions in an effort to meet this need:

1. To ensure that all stakeholders involved in the implementation of medical technologies in prehospital response for MCIs have a common understanding, it is recommended to provide an initial introductory inductive seminar addressed to all the partners involved (both medical and technical). This seminar should cover the basics of prehospital response procedures and how medical technologies assisting in the response are developed. Regular "refresher" sessions, in the format of knowledge-sharing, should also be held to ensure that everyone is up to date with the latest developments and changes. In this way any confusion regarding the terminology used and the scope of the project will be avoided as well as any gap in knowledge of both the medical and the technological partners involved will be bridged.
2. It is important to remember that the primary focus should always be on the activity itself, which is the prehospital response in MCI settings. The use of medical technologies should be seen as a means to enhance and improve the response rather than as an end in itself. It is recommended that a clear framework be established that outlines how medical technologies can be integrated into the prehospital response process and that KPIs are determined and monitored to assess the effectiveness of the technology.
3. To ensure effective integration of medical technologies in prehospital response for MCIs, it is recommended that the people leading similar projects have a combination of medical knowledge and experience in prehospital response for MCIs, as well as up-to-date knowledge of available technologies. This will enable them to effectively assess the potential of new technologies and ensure that they are integrated in a way that enhances the overall response. Close collaboration between medical and technical experts is also recommended to ensure that the translation of medical needs to technological solutions is not lost in stereotype assumptions.

## Conclusion

The NIGHTINGALE project, funded by the European Union Horizon 2020 research and innovation program, is a significant initiative that seeks to develop a comprehensive toolkit to support FRs involved in prehospital MCI response. The recent study conducted as part of this project shed light on various challenges when evaluating the impact of such toolkit on FRs’ performance, underscoring the importance of a more structured and collaborative approach to the identification and use of KPIs in the context of MCI response using technological tools, with a specific focus on addressing the lack of consistent terminology, prioritizing tool validation over direct measurement of processes, recognizing the context-dependent nature of benchmarking, and bridging the gap between the technological and medical worlds. These insights are crucial for enhancing the prehospital response to disasters and improving overall disaster management.

## Supplementary Information

Below is the link to the electronic supplementary material.

Supplementary file1 (DOCX 15 KB)

## Data Availability

No datasets were generated or analysed during the current study.
