# Supplementary material for: Key performance indicators and benchmarks in MCI prehospital response using technological tools: a qualitative study assessing the perception of practitioners and tool developers
Source: Eur J Trauma Emerg Surg. 2024 Aug 22;50(5):2273–9. doi: 10.1007/s00068-024-02627-3 (PMC11599289; doi:10.1007/s00068-024-02627-3)
Supplement: Supplementary file 1 — Supplementary file1 (DOCX 15 KB) [file 68_2024_2627_MOESM1_ESM.docx]

| **Category of NIT-MR tools** | **KPIs** | | | **User Satisfaction Rate %** |
| --- | --- | --- | --- | --- |
|  | **Time indicators** | **Process indicators** | **Output indicators** |  |
| Triage and Vital Signs devices. | x | x |  |  |
| Multi agency collaboration systems | x | x | x | x |
| Interoperability and data fusion |  | x |  | x |
| Command, control, and coordination management system | x |  | x | x |
| UAV | x | x | x |  |
| Damage Control and AI-based diagnosis and prognosis |  | x |  | x |

*Supplementary table 1*. Mapping of KPIs identified by workshop participants for the NIT-MR tools grouped in the different categories. Time indicators either reflected performance of the tool (e.g. “Measurement output < 30 seconds”) or the process supported by the tool (e.g. “Time of deployment from arrival on scene < 30 minutes”). Process indicators delineated the tool's assistance with a specific process in a binary fashion, offering either a positive or negative reflection (Yes/No), while output indicators referred to the accuracy in supporting a specific process (e.g. “Accuracy in mapping victim location > 90%”).
